# Characterization of Anticipatory Postural Adjustments in Lateral Stepping: Impact of Footwear and Lower Limb Preference

**DOI:** 10.3390/s21248244

**Published:** 2021-12-09

**Authors:** Yuri Russo, Dragan Marinkovic, Borislav Obradovic, Giuseppe Vannozzi

**Affiliations:** 1 Interuniversity Centre of Bioengineering of the Human Neuromusculoskeletal System (BOHNES), Department of Movement, Human and Health Sciences, University of Rome “Foro Italico”, Piazza Lauro De Bosis, 6, 00135 Rome, Italy; russo.yr@gmail.com (Y.R.); marinkovic@uns.ac.rs (D.M.); 2 Faculty of Sport and Physical Education, University of Novi Sad, Lovcenska 16, 21000 Novi Sad, Serbia; bobradovic@uns.ac.rs

**Keywords:** piezo-resistive sensors, force plate, APA, biomechanics, posture, step, shoe, footwear, laterality

## Abstract

Lateral stepping is a motor task that is widely used in everyday life to modify the base of support, change direction, and avoid obstacles. Anticipatory Postural Adjustments (APAs) are often analyzed to describe postural preparation prior to forward stepping, however, little is known about lateral stepping. The aim of the study is to characterize APAs preceding lateral steps and to investigate how these are affected by footwear and lower limb preference. Twenty-two healthy young participants performed a lateral step using both their preferred and non-preferred leg in both barefoot and shod conditions. APA spatiotemporal parameters (size, duration, and speed) along both the anteroposterior and mediolateral axes were obtained through force plate data. APAs preceding lateral stepping showed typical patterns both along the anteroposterior and mediolateral axis. RM-ANOVA highlighted a significant effect of footwear only on medio-lateral APAs amplitude (*p* = 0.008) and velocity (*p* = 0.037). No differences were found for the limb preference. APAs in lateral stepping presented consistent features in the sagittal component, regardless of limb/shoe factors. Interestingly, the study observed that footwear induced an increase in the medio-lateral APAs size and velocity, highlighting the importance of including this factor when studying lateral stepping.

## 1. Introduction

Anticipatory Postural Adjustments (APAs) are postural movements that precede most voluntary movements and are produced to create favorable conditions for the latter [1]. APAs are specific for the movement they precede and are modulated based on afferent information and environmental factors [2,3]. Their role is two-fold: to maintain the body’s equilibrium and to facilitate/control the movement [1,4]. In lower limb movements, in which balance must be finely controlled to avoid falls, APAs exert a control over the body’s center of mass (CoM) by decoupling the center of pressure (CoP)-CoM movements [5]. In forward step/gait initiation for instance, APAs move the CoP toward the stepping leg and backwards, while the CoM moves toward the stance leg, and forward [5,6]. Each of these components of the APAs that precede a step, the mediolateral (ML) and the anteroposterior (AP) one, have been linked to different functions: the ML component has been classically associated with stability [7], whereas the AP one has been associated with performance [8]. Our understanding of APAs in lower limb movements primarily comes from studies that both characterized postural preparation preceding forward step/gait initiation, and investigated this task in special populations [5,9,10,11,12,13]. However, little attention has been directed toward steps in non-forward directions [14,15,16], even though between 10% and 50% of steps performed everyday fall within this category [17]. Despite their paucity, studies investigating non-forward steps (e.g., diagonal, lateral stepping) relevantly contributed to our understanding of postural control, highlighting how the performance of these tasks may unveil biomechanical differences between pathological and healthy populations [18].

Lateral stepping is often used by people as a recovery strategy to regain balance [19]. In fact, by modulating the features of the base of support, individuals attempt to adopt a more stable posture where the CoM is repositioned far from the edges of the base of support. Consequently, most research related to lateral stepping investigated its preparation and execution in specific paradigms, in which participants had to take a step as fast as possible, or in response to a perturbation in an attempt to restore balance [20,21,22]. Nevertheless, lateral steps performed under perturbation conditions are different compared to voluntary ones even when performed as fast as possible [23]; specifically, lateral reactive steps tend to be faster [24] and larger compared to voluntary ones [23]. Therefore, considering the close relationship between preparation and execution of the step [13,25,26], results obtained in reactive or velocity constrained paradigms cannot be generalized to self-paced voluntary steps. To our knowledge only a few studies investigated the execution of voluntary lateral stepping [15,23,24,27], although none of them investigated this task in unconstrained conditions. 

Additionally, we know that several factors influence step initiation in the forward direction (e.g., age, existing pathologies, sensory information etc. [2,3,28,29,30]), significantly modifying APAs patterns and characteristics. For instance, footwear and limb preference play a relevant role, especially when considering everyday life conditions [31,32,33]. Footwear is present in most of the steps people take every day, providing sanitary and mechanical protection, yet at the same time modifying body kinematics [34,35,36,37]. Research has shown that footwear induces alteration of gait either by altering the ankle range of motion [38] or by influencing somatosensory information [39,40]. Since APAs are scaled based on sensory information [2,3], they are modified when a step is taken in a shod condition compared to barefoot conditions [32,33]. Additionally, limb preference defined as the preferential use of a limb in voluntary motor actions [41] is demonstrated to impact tasks of posture and gait. In gait studies, lower limb distinction is generally made according to the role of the limb as either supportive (non-dominant) or propulsive (dominant) [31,41]. Although gait is by nature symmetrical, several studies found significant biomechanical differences between the legs [41,42]. Contrarily to locomotion, stepping is an asymmetric task in which each limb plays a different role, therefore, starting a step either with a preferred or a non-preferred leg may impact not only APAs but also characteristics of the first step during forward step initiation [31,43]. In lateral stepping, due to the plane of body motion, limb preference may play a more important role; in this case, the direction of the step does not just rely on personal preferences but also on other factors such as environmental demands (e.g., avoiding objects present in/coming from a defined direction). As shown, only recently has the forward step literature taken footwear and limb preference into account, and despite the existing need for further studies, our understanding of ecological stepping has moved forward. However, due to the lack of research on non-forward steps, the impact of footwear and leg dominance on lateral stepping has currently not been examined.

Thus, the aim of this study was to characterize the biomechanics of APAs in voluntary lateral stepping, taking into account the role of both footwear and limb preference in postural preparation. We hypothesize that footwear would induce an increase in the postural preparation prior to lateral stepping, compared to the barefoot condition, especially along the ML axis. In addition, we expect a significant effect of limb preference on APAs parameters, with larger and faster APAs produced when stepping with the preferred leg.

## 2. Materials and Methods

### 2.1. Study Design

This observational study was performed according to participants repeated-measure design. All the participants performed a lateral stepping task under four different experimental conditions: (i) barefoot/stepping with the dominant leg; (ii) barefoot/stepping with the non-dominant leg; (iii) shod/stepping with the dominant leg; (iv) shod/with the non-dominant leg. The order of the conditions was randomized across the subjects.

### 2.2. Participants

Participants were recruited among students and staff of the University of Rome “Foro Italico”. Inclusion criteria were: (i) no history of neurological or musculoskeletal disorders; (ii) no clinical conditions that could impair balance; (iii) a BMI between 18.5 and 29.9 kg/m^2^; (iv) that participants were aged between 18 and 45 years old. Exclusion criterion was the frequent practice (>3 h per week) of barefoot sports. After the explanation of the experimental protocol, each participant provided their informed consent in writing, in accordance with the Declaration of Helsinki. The study was approved by the Institutional Review Board of University of Rome “Foro Italico”.

### 2.3. Instruments and Outcome Measures

The acquisition of the kinetic data was performed using a force plate (40 × 40 cm Bertec Corp, Columbus, OH, USA) embedding piezo-resistive sensors, which was mounted flush to the floor. Ground Reaction Force (GRF) and CoP data were collected at a sampling frequency of 1000 Hz. The force plate was unloaded and reset after each trial to prevent signal drift. APAs were measured, both along the ML and AP direction, using the CoP trajectories. In line with previous work [15,32,33], spatiotemporal parameters of APAs (i.e., amplitudes, durations and velocities) were used to characterize the task and to assess the effect of limb dominance and footwear on postural preparation.

### 2.4. Experimental Protocol

Participants were asked to stand on the force plate, in a comfortable position, with both arms resting at their sides. They were asked to distribute their body weight equally between the two feet, which were hip-distance apart. For each person, after the familiarization period, the comfortable base of support was marked on the force plate for the sake of standardization. After receiving a verbal command from the experimenter (“whenever you are ready”), the participants initiated the lateral step whenever they preferred and at their comfortable speed. The protocol was performed both in barefoot (BF) and shod (SH) conditions. The participants were asked to bring their own cross-training shoes [44]. To reduce the variability associated with the footwear; other types of footwear were not allowed for the experiment (e.g., court shoes, loafers). In addition, participants were asked to initiate lateral steps both with their dominant and non-dominant limb. The limb dominance was determined by asking participants what leg they would kick a ball with [45,46,47]. Each volunteer performed 28 trials in total (7 trials in each condition). 

### 2.5. Data Analysis

**Figure 1:**
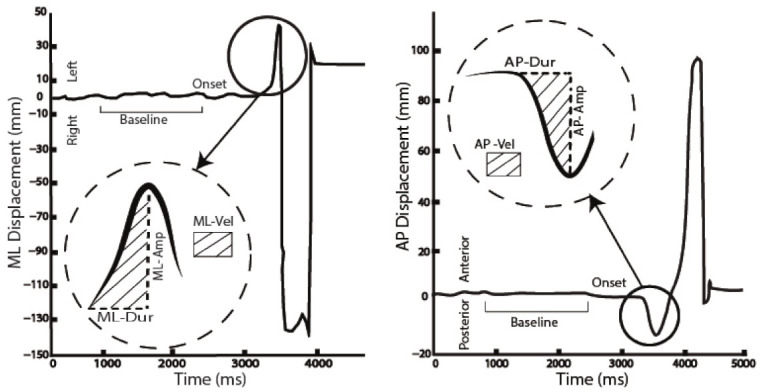
Anticipatory Postural Adjustments (APAs) parameters measured during lateral step (shod condition) in a randomly selected participant. ML (**left**) and AP (**right**) trajectories of the Center of Pressure (CoP) during APAs are represented. Examples of CoP baseline, APAs onset, APAs peak and CoP mean velocity are reported. The six APA parameters are included: Amplitude (ML-Amp; AP-Amp), Duration (ML-Dur; AP-Dur) of time to peak magnitude from onset, and Velocity (ML-Vel; AP-Vel).

CoP was low-pass filtered at 10 Hz (Butterworth, 4th order) [48]. Baseline values of AP and ML CoP displacements were identified within the first 2.5 s at the beginning of each trial when subjects were asked to stand still (see Figure 1). ML and AP APA onsets were defined as the instants when the first-time derivative of the relative CoP displacement exceeded three standard deviations from the baseline. Due to the laboratory reference system and the orientation of participants, ML and AP peak were identified using different approaches. Specifically: (i) ML APA peak was identified as the ML CoP maximum within 1 s after ML APAs onset; whereas (ii) AP APA peak was identified as the AP CoP minimum within 1 s after AP APAs onset (Figure 1). The reference system was adjusted across the conditions to allow the identification of the instants as described above; particularly with the ML axis always pointed toward the stance leg [15,33]. The following parameters were computed (see Figure 1): APA-ML and -AP durations, amplitudes, and CoP velocity. APAs durations were calculated as the time difference between the identified APAs peak and APAs onset. APAs amplitudes were defined as the absolute value of the spatial difference between the CoP coordinates at APAs peak and at baseline. Lastly, CoP velocities were estimated as the ratio between APAs amplitude and APAs duration. The analysis was semi-automatically performed using customized software written in MatLab R2018b (The MathWorks, Natick, MA, USA).

### 2.6. Statistical Analysis

A Shapiro–Wilk test was performed to assess normality of data distribution. In case of non-normal distribution, data were transformed using the Tukey’s ladder of powers. Mean and standard deviations were calculated for each parameter. The parameters were analyzed with a 2 × 2 Repeated Measure Analysis of Variance (RM-ANOVA) with Footwear (Shod vs. Barefoot) and Side (Preferred vs. Non-Preferred) as within factors. Level of significance was set to α = 0.05. The analysis was performed using SPSS 25 (IBM Statistics, Chicago, IL, USA).

## 3. Results

Twenty-five participants were recruited in the study. A participant was excluded as they brought the wrong footwear type, and two participants were excluded due to technical problems during the recordings. Twenty-two participants (11 females, 11 males; age 26.0 ± 5.4; height 1.69 ± 0.08 m; body mass 61.6 ± 9.9 kg; BMI 21.54 ± 2.37 kg/m^2^) successfully completed the experimental protocol. 

CoP trajectories presented specific characteristics describing postural preparation prior to lateral stepping. In barefoot condition, here representing the baseline condition, ML APAs showed a mean amplitude of 12.4 mm and an average duration of 417 ms, whereas AP APAs amplitude showed a mean amplitude of 12.5 mm and an average duration of 559 ms. 

RM-ANOVA revealed a main effect of the footwear condition (shod vs. barefoot, see Table 1) that entailed significant increases both in ML amplitude (F_(1,21)_ = 8.453, *p* = 0.008) and ML velocity (F_(1,21)_ = 4.989, *p* = 0.037). Contrarily, no significant differences were found between sides (Preferred vs. Non-Preferred). Furthermore, no significant interactions condition X side were detected for either ML or AP parameters.

## 4. Discussion

The objective of the study was two-fold: (i) to describe the APAs related to a voluntary lateral step; and (ii) to investigate the effect of footwear and limb dominance on the postural preparation preceding this task. Our results provide a biomechanical characterization of APAs in voluntary lateral steps, highlighting the importance of considering environmental factors, such as footwear, when investigating the postural preparation prior to a lower-limb movement.

**Figure 2:**
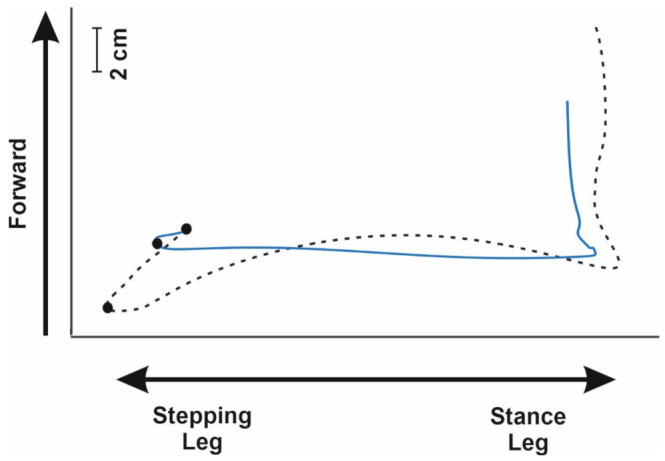
The figure illustrates, to allow a clear comparison, CoP trajectories associated with postural preparation prior to a lateral step (light blue line) and a forward step (black broken line) in the same participant; data of the forward step initiation was collected, analyzed and published as part of another study [33]. The trials were performed in barefoot condition. The black dots highlight the start and end of APAs for the two tasks.

The results of the present study support and extend previous findings suggesting that, in lateral stepping, postural preparation is shorter in duration and smaller in amplitude compared to forward stepping [15]. To better highlight this circumstance, the 2D trajectory of a lateral stepping trial is reported in Figure 2, highlighting the comparison with a forward stepping trial of the same participant taken from an available dataset [33]. 

The graphical comparison of the two 2D trajectories more than justifies the observed differences in amplitudes of both the AP and ML axes.

According to Patla and co-workers [21], the duration of the weight shifting toward the standing limb (observed in the time-behavior of the vertical GRF) in lateral steps is reduced. This strategy leads to the generation of adequate angular velocities and accelerations which facilitate the movement in the desired direction through pivoting around the standing leg. In contrast, Sparto and colleagues [27] observed that young people, compared to old ones, tend not to use weight shifting strategies prior to lateral stepping, so do have shorter step latencies (i.e., time period between postural preparation and execution) and faster step execution. In forward stepping, APAs are considered to be absent when the produced CoP displacement prior to the step is lower than 5 mm [49]. In this study, healthy adults produced small, yet consistent, ML shifting of the CoP toward the stepping leg, which represents a strategy people use to move the center of mass toward the stance leg, thus increasing stability (i.e., weight shifting) [4]. From this perspective, the present study supports Patla’s results, suggesting that in lateral steps postural preparation still plays an important role in stabilizing the body and creating the conditions for the movement by regulating the displacement of the CoP and, therefore, modulating the acceleration of the CoM during the movement similarly to forward stepping [5]. In addition, we found that during lateral step preparation, there is a small but consistent AP displacement of the CoP, which is backward directed, that was not affected by footwear or limb preference. Our interpretation is that people tend to slightly lean forward prior to a voluntary lateral step, producing the mentioned CoP displacement, to control body balance with the mid/forefoot. AP APA in lateral step was not investigated [21,27] nor reported [15] by previous studies; however, it is possible that Inaba and co-workers [15] could not consistently observe this modest APA due to its variability and the small sample size of the study (6 participants). Indeed, further research focusing on body kinematics is required to endorse or confute this hypothesis.

One of the novelties of this study is indeed the investigation of footwear effects in lateral steps. Previous studies on forward step initiation highlighted that shoes do not impact postural preparation along the AP axis [32,33]; consistently, our results showed no significant effects of footwear along the AP direction. A possible explanation is that, although shoes modify dynamic postural control and foot stability [35,36,37,50,51], they do not influence the mechanism underlying the AP CoP displacement as much (i.e., ankle dorsiflexion) [13,15,52]. On the contrary, and in line with the literature on forward step [32,33], footwear significantly affected APAs along the ML direction. APAs are known to be modulated by the central nervous system based on environmental factors [53,54,55] as well as tactile, kinesthetic, and proprioceptive information [2,3,56]. The literature shows that footwear may represent a constraint for ankle and foot joints [35,37,38] and may also interfere with afferent information gathered by the feet soles, due to the interposition of cushioning elements (e.g., midsole). In addition, larger postural preparation along the ML direction may result in smaller velocities and accelerations of the body during the movement [21,27]. In keeping with that, we speculate that the central nervous system, due to the presence of cross-training shoes, would aim to produce more stable conditions for the movement, which are reflected by the larger CoP displacement observed in footwear condition (i.e., wider weight shifting). Interestingly, healthy participants did not modify the total duration of APAs, which together with the increase in their amplitude, led to faster CoP displacement when shoes were worn. Indeed, increasing the amplitude and the velocity of APAs, without altering their duration, imply the production of larger forces, which in special populations (e.g., elderly, stroke) might be a counter-productive strategy in terms body balance. Therefore, it is plausible that these populations may opt for different strategies, as for example, one in which the increase in APA amplitude goes along with the increase in APAs duration rather than its velocity. This aspect could be certainly explored in future work aimed at translating this knowledge in clinical gait analysis contexts.

Contrarily to our initial hypothesis, no significant differences were found in postural preparation prior to lateral steps, between preferred and non-preferred limb, for the APAs parameters considered (i.e., duration, amplitude and velocity). To our knowledge, this is the first study investigating limb preference in APAs before a voluntary lateral step. Previous articles have shown that postural preparation prior to gait initiation may be affected by limb preference [31,43]. In forward stepping, however, weight-shifting and movement performance mainly rely on different components of APAs, the first of which occur along the ML axis and the latter along the AP [7,8]. In lateral stepping, weight shifting and motor performance are more intertwined since more weight-shifting implies less angular velocity/acceleration, which in the end would result in slower step execution [21]. We speculate that, in young healthy people, the absence of significant differences between lateral step preparation in preferred and non-preferred limb may be the result of a trade-off between stability and performance. Further studies could investigate whether this symmetry is consistent in other populations (e.g., elderly, fallers, people with Parkinson’s disease), or rather disrupted by the underlying condition.

### Study Limitations

In the shod condition, the experiment was carried out using only one type of footwear, and therefore the results of this study cannot be used to make generalizations regarding footwear other than cross-training shoes (e.g., running, court, sandals). Whole-body kinematics and muscular activities of the trunk/lower limb were not measured in this experiment; further studies investigating this task might also include such measurements to fully characterize lateral stepping.

## 5. Conclusions

The characterization of the lateral stepping provided in this study extends beyond the previous literature, highlighting not only the relevance of the medio-lateral control, which in lateral step is crucial for both balance and motor performance, but also showing for the first time the presence of a small but consistent antero-posterior APA. The inclusion of the footwear as an experimental condition allowed us to observe how shoes modify the postural preparation parameters, which together with the absence of difference between preferred and non-preferred limb, point out the potential for the translation of this knowledge in clinical contexts.

## Figures and Tables

**Table 1:** APA parameters values (mean ± std. dev.) for the participants, divided between experimental conditions. Main effects for Condition (Barefoot vs. Shod; *p* < 0.05) are indicated in bold. * indicates difference with respect to the barefoot condition.

APA		Preferred Limb		Non-Preferred Limb	
		Barefoot	Shod	Barefoot	Shod
Duration (ms)	ML	420 ± 55	443 ± 80	413 ± 104	432 ± 67
	AP	553 ± 149	538 ± 127	564 ± 141	531 ± 135
Amplitude (mm)	**ML**	**12.3 ± 5.9**	15.8 ± 7.5 *	**12.5 ±6.9**	14.6 ± 5.8 *
	AP	12.7 ± 4.2	12.5 ± 5.4	12.2 ± 3.9	11.6 ± 4.2
Velocity (m/s)	**ML**	**0.030 ± 0.015**	0.037 ± 0.016 *	**0.032 ± 0.016**	0.036 ± 0.015 *
	AP	0.025 ± 0.008	0.024 ± 0.007	0.023 ± 0.007	0.024 ± 0.008

## Data Availability

The data presented in this study could be made available upon request to the corresponding author.
